# A Computer Vision-Based Yoga Pose Grading Approach Using Contrastive Skeleton Feature Representations

**DOI:** 10.3390/healthcare10010036

**Published:** 2021-12-25

**Authors:** Yubin Wu, Qianqian Lin, Mingrun Yang, Jing Liu, Jing Tian, Dev Kapil, Laura Vanderbloemen

**Affiliations:** 1 Institute of Systems Science, National University of Singapore, Singapore 119615, Singapore; e0703350@u.nus.edu (Y.W.); e0703470@u.nus.edu (Q.L.); e0703376@u.nus.edu (M.Y.); e0703435@u.nus.edu (J.L.); 2 One Wellness Pte Ltd., Singapore 188033, Singapore; dev@onewellness.com.sg; 3 College of Health Sciences, VinUniversity, Hanoi 10000, Vietnam; laura.v@vinuni.edu.vn; 4 Faculty of Medicine, Imperial College London, London SW7 2AZ, UK

**Keywords:** yoga pose grading, skeleton extraction, contrastive learning, yoga pose classification, deep learning

## Abstract

The main objective of yoga pose grading is to assess the input yoga pose and compare it to a standard pose in order to provide a quantitative evaluation as a grade. In this paper, a computer vision-based yoga pose grading approach is proposed using contrastive skeleton feature representations. First, the proposed approach extracts human body skeleton keypoints from the input yoga pose image and then feeds their coordinates into a pose feature encoder, which is trained using contrastive triplet examples; finally, a comparison of similar encoded pose features is made. Furthermore, to tackle the inherent challenge of composing contrastive examples in pose feature encoding, this paper proposes a new strategy to use both a *coarse triplet example*—comprised of an anchor, a positive example from the same category, and a negative example from a different category, and a *fine triplet example*—comprised of an anchor, a positive example, and a negative example from the same category with different pose qualities. Extensive experiments are conducted using two benchmark datasets to demonstrate the superior performance of the proposed approach.

## 1. Introduction

Yoga pose grading aims to quantitatively evaluate yoga poses so that it can realize yoga pose recognition (how a yoga pose is performed) and evaluate pose quality (how well a yoga pose is performed) [1,2]; which can distinguish different movements by analyzing pose characteristics. The most important aspect of yoga exercise is to do it correctly, since any wrong position can be counterproductive and possibly lead to injury [3,4,5]. However, not all users have access to a professional instructor. Many yoga beginners could only learn yoga by self-study, such as mechanically copying from a recorded yoga video or remotely watching a live yoga session. Consequently, they have no way of knowing if their pose is good or poor without the help of the instructor. Therefore, automatically evaluating yoga poses is critical to the recognition of yoga poses and in providing suggestions to alert learners [6].

There are various types of artificial intelligence-based solutions for yoga pose analysis that have been developed in the literature, including (i) *the wearable device*-based approach [7,8], (ii) *the Kinect*-based approach [9,10,11], and (iii) *the computer vision*-based approach.

First, *wearable device*-based approaches usually require attaching sensors to each joint of the human body during yoga exercise. Wu et al. proposed a pose recognition and quantitative evaluation approach [7]. A wearable device with eleven *inertial measurement units* (IMUs) is fixed onto the human body in order to measure yoga pose data. Then, the artificial neural network and fuzzy C-means are combined to classify the input pose into a category. In addition, the angular differences between nonstandard parts (e.g., the yoga student) and the standard pose model (e.g., the yoga teacher) are calculated to guide yoga learners. Puranik et al. proposed a wearable system [8] where a wrist subsystem is used to monitor a pose with the help of a flex sensor, and a waist subsystem is built to monitor the pose with the use of a flex sensor. However, such solutions are impractical for long-term applications due to their maintenance concerns.

Second, *Kinect*-based approaches deploy the Kinect device to extract features. Chen et al. captured the yoga learner’s body map and extracted the body contour [9]. Then, a fast skeletonization technique was used as a human pose feature for yoga pose recognition. Trejo and Yuan presented a yoga pose classification approach by employing the KinectV2 camera and the Adaboost classifier algorithm for recognizing six poses [10]. Islam et al. presented a yoga pose recognition method that leverages fifteen keypoints detected from Kinect camera images and uses pose-based matching for pose recognition [11]. However, the depth sensor-based camera required in these solutions may not be always available for users.

Third, *computer vision*-based approaches use non-invasive computer vision techniques to extract pose characteristics and perform pose analysis, as reviewed in Section 2. They are more suitable for amateur training and home exercise. Many studies have begun to examine how to utilize human pose analysis techniques in the field of intelligent sports learning since the invention of human pose analysis techniques [12].

Computer vision-based yoga pose grading is a difficult task due to the following challenges. The first challenge is due to the lack of a yoga pose grading benchmark as image-level annotation is expensive; hence, the supervised representation learning might not be feasible. The second challenge lies in the fundamental difference between the learner’s pose image and the standard pose image. The aggregated features using multiple deep features from the pre-trained models might be more robust than a single type of feature [13]. In addition, human body skeleton information might be robust to handle this diversity. To tackle these challenges, the contrastive learning technique [14,15,16] is a potential solution. Its key idea is to conduct a discriminative learning approach to learn encoded feature representations, in which similar sample pairs remain close together, whereas different sample pairs remain widely apart. It has been successfully verified in many computer vision tasks such as image classification [17] and human activity recognition [18,19].

Motivated by this, a computer vision-based yoga pose grading approach using contrastive skeleton feature representations is proposed in this paper. The following are the main contributions of this paper:

- To tackle the challenge of variation between the learner’s pose image and the standard pose image, contrastive learning is introduced in this paper to develop a yoga pose grading approach that uses contrastive skeleton feature representations instead of diverse and complicated backgrounds in the images. The proposed approach is able to learn discriminative features from human skeleton keypoints for yoga pose grading, as verified in our experimental results.
- To tackle the challenge of the establishment of contrastive examples used for discriminative feature learning, a novel strategy is proposed in this paper to compose the contrastive examples using both the *coarse triplet example*, which consists of an anchor, a positive example from the same category, and a negative example from a different category, and the *fine triplet example*, which consists of an anchor, a positive example, and a negative example from the same category with different pose qualities.

The rest of this paper is organized as follows. Section 2 provides a brief review of the existing research works in yoga pose classification and yoga pose grading. Then, the proposed yoga pose grading approach using contrastive skeleton feature representations is presented in Section 3, and then evaluated in extensive experiments in Section 4. Limitations and future studies are also provided in Section 4. Finally, this paper is concluded in Section 5.

## 2. Related Works

This section provides a brief review of related computer vision-based research works with a focus on (i) yoga pose classification [20,21,22,23,24,25,26,27,28] and (ii) yoga pose grading [29,30,31,32], as summarized in Table 1.

### 2.1. Yoga Pose Classification

Recently, deep learning has achieved an impressive performance in addressing the yoga pose classification task due to its powerful feature learning capability. Yadav et al. proposed a hybrid deep learning framework where the *convolutional neural network* (CNN) layer is used in each frame to extract features from human body keypoints returned by OpenPose [33], followed by the *long short-term memory* (LSTM) layers performing temporal learning [20]. Maddala et al. proposed to integrate joint angular movements along with the joint distances in a spatiotemporal color-coded image, which is further analyzed using a CNN model [21]. To address the privacy issue in the camera-based solution, Gochoo et al. proposed a privacy-preserving yoga pose recognition by utilizing a deep CNN and a low-resolution infrared sensor [22]. The OpenPose-based skeleton keypoint extraction and the CNN model were also studied in [23]. Special attention was paid to applying a rule-based classification in order to detect fall risk during yoga exercise in [24]. A benchmark dataset for fine-grained yoga pose classification and several CNN baselines are provided in [25]. Other examples of deep learning-based yoga pose classification include the image-based CNN model and transfer learning [26,27], and the three-dimensional CNN model for yoga videos [28].

### 2.2. Yoga Pose Grading

In contrast to the objective of yoga pose classification to infer the yoga pose class label, yoga pose grading aims to automatically quantify how well people perform yoga actions. Despite the fairly popular studies on yoga pose classification, there are not many works on yoga pose grading. Patil et al. proposed to identify yoga pose variations between different persons by comparing the similarity between the *speeded up robust feature* (SURF) extracted from the input pose images [29]. Chen et al. proposed to capture the user body map, and then apply the skeleton to extract the human body feature points to identify the correct pose [30]. Chaudhari et al. used the domain knowledge of five yoga poses to build a system that delivers clear feedback to the practitioner for them to appropriately practice yoga postures. They employed a CNN model to identify yoga poses as well as a human-joint localization model to detect flaws in the pose [31]. Kale et al. built a knowledge base of twenty-one poses for examining the skeletal stream of specialists to see if there were any differences [32].

### 2.3. Motivation and Research Challenge

Despite the fairly popular studies in yoga pose classification, there is a lack in yoga pose grading research, except the works in [29,30,31,32]. The limitations of existing works lie in two aspects:

- First, it is a challenge to rely on the whole pose image for pose grading due to the fundamental difference between the learner’s pose image and the standard pose image. To address this, the proposed approach exploits the skeleton keypoints from the pose image, or more specifically, the discriminative features that are learned from the contrastive skeleton feature representations. This is in contrast to what the whole pose image is used in [29].
- Second, the domain knowledge is required to define customized rules for specific yoga pose grading. It is difficult for them to handle new types of yoga poses. For example, the methods in [30,31,32] require the domain knowledge to define the rules in order to evaluate yoga poses by checking characteristics (e.g., positions or angles) of the skeleton keypoints of various yoga postures. To address this, the proposed approach relies on machine learning methods in order to provide general yoga grading without the need for additional domain knowledge.

In summary, to tackle these challenges, a pose grading approach using contrastive skeleton feature representations is proposed in this paper.

## 3. Proposed Approach

**Figure 1:**
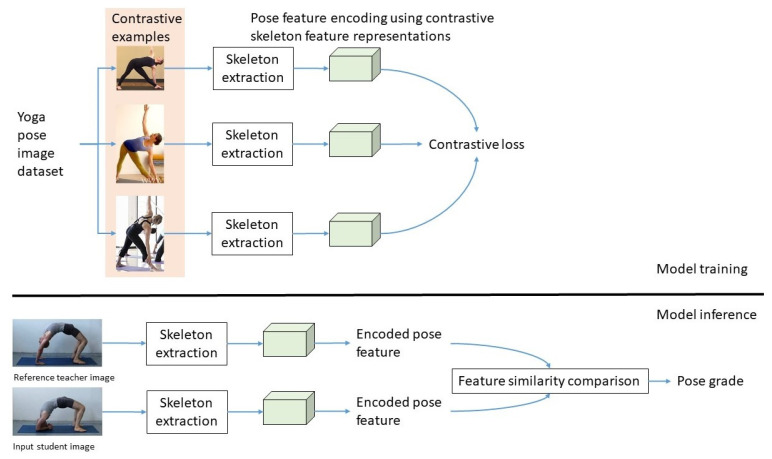
A conceptual overview of the proposed yoga pose grading framework. The model training process consists of three key components: (i) construction of contrastive examples, (ii) skeleton extraction, (iii) pose feature encoding using contrastive skeleton feature representations. The model inference process consists of (i) skeleton extraction, (ii) pose feature encoder, and (iii) feature similarity comparison. Both skeleton extraction and pose feature encoder are the same in these two processes.

The objective of the proposed yoga pose grading approach is to input two yoga pose images from the learner and the coach, respectively, and then extract the human skeleton keypoints and feed them into the pose feature encoder. Finally, the feature similarity between them is calculated in order to obtain a pose grade. As illustrated in Figure 1, the proposed framework consists of a model training process and a model inference process. More specifically, the model training process consists of three key components: (i) construction of contrastive examples, (ii) skeleton extraction, (iii) pose feature encoding using contrastive skeleton feature representations. The model inference process consists of (i) skeleton extraction, (ii) pose feature encoder, and (iii) feature similarity comparison. All of these components are described in the following sections in detail.

### 3.1. Construction of Contrastive Examples

The proposed framework exploits the contrastive learning concept, which applies a weight-sharing neural network on multiple inputs. This is a natural tool to compare various pose images. To learn effective discriminative representations, the composition of multiple contrastive data is crucial in defining the contrastive prediction tasks. For that, we exploit the triplet example [34] in this work. The idea is to learn discriminative feature embedding representations where similar features are projected onto the nearby region, whereas dissimilar features are projected far away from each other.

**Figure 2:**
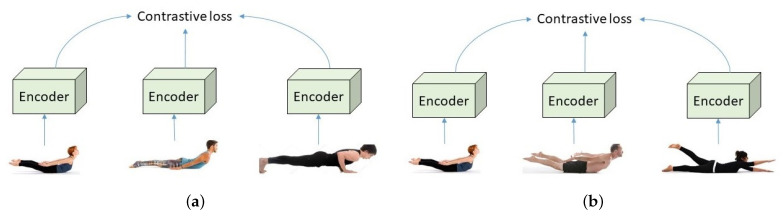
A comparison between (**a**) the *coarse triplet example* and (**b**) the *fine triplet example*. The coarse triplet example consists of one anchor from *Salabhasana*, one positive example from *Salabhasana*, and one negative example from a different category such as *Chaturanga Dandasana*. The fine triplet example consists of three examples from the same category such as *Salabhasana*; however, they have different pose grades: *high*-quality, *medium*-quality, *low*-quality (for the images from the left to the right, respectively).

To be more specific, we propose to use both the *coarse triplet example*—comprised of an anchor, a positive example from the same category, and a negative example from a different category, and the *fine triplet example*—comprised of an anchor, a positive example, and a negative example from the same category with different pose qualities. To illustrate the difference between these two types of triplet examples, a few examples are presented in Figure 2.

### 3.2. Skeleton Extraction

Due to the fact that some yoga poses are too complicated to be captured from a single point of view, the utilization of skeleton keypoints of the human targets in the pose images may be more suited for analyzing various poses than the whole pose image. In view of this, the proposed framework exploits the human skeleton keypoints in yoga pose grading instead of analyzing the whole pose image that is usually difficult due to diverse backgrounds and human appearance.

In this paper, we adopt Mediapipe [35], which utilizes a state-of-the-art machine learning model BlazePose [36] for skeleton keypoint extractions. It detects human body parts and tracks keypoints on these body parts. Each of these keypoints represents a two-dimensional coordinate that yields values in the range of (0,1) corresponding to the position of the pixel in the image, normalized with respect to image width and height. The implementation details are provided as follows. The *static_image_mode* is set to *True* as we process the single pose image as the input, the *minimum_detection_confidence* is set to the default value 0.5, and the *model_complexity* is set to 2 to obtain the most accurate keypoint results. After Mediapipe is applied to the input pose image, 33 keypoints of the human body are detected in one pose image. Each keypoint of the human body has two coordinate values; therefore, an image contains (2,33) coordinate data values that will be used in the following pose feature encoder.

### 3.3. Pose Feature Encoding Using Contrastive Skeleton Feature Representations

**Figure 3:**
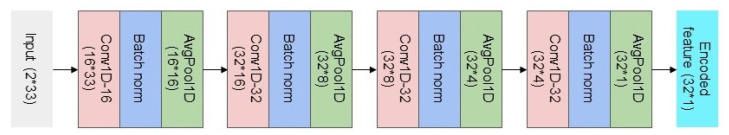
The detailed network architecture of the pose feature encoder that is used in the proposed framework.

The proposed approach aims to learn the discriminative representations by maximizing the agreement between similar yoga pose images via a contrastive loss in the latent feature space. It consists of the following key components:

- A neural network encoder (denoted as f(·)) that extracts representation vectors from input contrastive data examples. It maps representations to the space where contrastive loss is applied. The detailed network architecture is illustrated in Figure 3. The proposed encoder takes the introduced skeleton points as the input, and then it adopts a sequence of *Conv1D* layers, where the numbers of filters are 16,32,32,32; each filter has the same kernel size of 15. The batch normalization and average pooling are applied after each *Conv1D* layer. Finally, the encoded feature is obtained with a dimension of 32.
- When the *coarse triplet example* is used, the encoder takes a triplet example $x_{a}$, $x_{p}$, and $x_{n}$ as the input. These three images are processed to extract their respective skeleton points $s_{a}$, $s_{p}$, and $s_{n}$, each of which has a size of (2,33). Then, they are further processed by a weight-shared encoder network f(·) to obtain their respective features $z_{a}$, $z_{p}$, and $z_{n}$. A triplet contrastive loss is defined as follows [34]:
  (1) $$L\left(z_{a},z_{p},z_{n}\right)=\max\left(∥z_{a}-z_{p}{∥}^{2}-{∥z_{a}-z_{n}∥}^{2}+\alpha_{c},0\right),$$
  where $\alpha_{c}$ is a margin between positive and negative examples.
- On the other hand, when the *fine triplet example* is used, the encoder takes a triplet example $x_{h}$, $x_{m}$, and $x_{l}$ as the input, all of which are from the same category but are of *high*-quality, *medium*-quality, and *low*-quality, respectively. These three images are processed to extract their respective skeleton points $s_{h}$, $s_{m}$, and $s_{l}$, each of which has a size of (2,33). Then, they are further processed by a weight-shared encoder network f(·) to obtain their respective features $z_{h}$, $z_{m}$, and $z_{l}$. A triplet contrastive loss is defined as follows:
  (2) $$L\left(z_{h},z_{m},z_{l}\right)=\max\left(∥z_{h}-z_{m}{∥}^{2}-{∥z_{h}-z_{l}∥}^{2}+\alpha_{h},0\right)+\max\left(∥z_{l}-z_{m}{∥}^{2}-{∥z_{l}-z_{h}∥}^{2}+\alpha_{l},0\right),$$
  where $\alpha_{h}$ and $\alpha_{l}$ are the margins when the *high*-quality example and the *low*-quality example are used as anchors, respectively.

In the model training, every batch consists of the same number of coarse triplet examples and fine triplet examples. Then, (1) and (2) are combined to form the final loss to supervise the model training as follows:

(3) $$\begin{array}{ccc} L & = & {\mathrm{AVG}}_{coarse}\left(\max\left(∥z_{a}-z_{p}{∥}^{2}-{∥z_{a}-z_{n}∥}^{2}+\alpha_{c},0\right)\right)\\ \; & \; & \;+5*{\mathrm{AVG}}_{fine}\left(\max\left(∥z_{h}-z_{m}{∥}^{2}-{∥z_{h}-z_{l}∥}^{2}+\alpha_{h},0\right)+\max\left(∥z_{l}-z_{m}{∥}^{2}-{∥z_{l}-z_{h}∥}^{2}+\alpha_{l},0\right)\right), \end{array}$$

where ${\mathrm{AVG}}_{coarse}\left(\cdot \right)$ and ${\mathrm{AVG}}_{fine}\left(\cdot \right)$ represent the average loss calculated using the coarse triplet examples and the fine triplet examples in the batch, respectively. In addition, the loss that is obtained from the fine triplet examples is further multiplied by a factor of 5 in this combination (3), as the fine triplet examples are treated as more important in the model training.

### 3.4. Inference

The model inference process consists of (i) skeleton extraction, (ii) pose feature encoder, and (iii) feature similarity comparison. The skeleton extraction and the pose feature encoder are the same as those used in the model training process. Given two input yoga pose images from the student and the teacher (denoted as $x_{s}$, and $x_{t}$, respectively), extract the human skeleton keypoints and feed them into the pose feature encoder, before finally calculating the feature similarity between their encoded features $z_{s}$ and $z_{t}$ m to obtain a pose grade as follows:

(4) $$Grade\left(z_{s},z_{t}\right)=\frac{z_{s}^{T}z_{t}}{\left|\right|z_{s}\left|||\right|z_{t}\left|\right|},$$

which calculates the dot product between the $L_{2}$ normalized $z_{s}$ and $z_{t}$ (i.e., cosine similarity).

## 4. Results

### 4.1. Dataset

Two benchmark datasets are used in our experiments.

**Figure 4:**
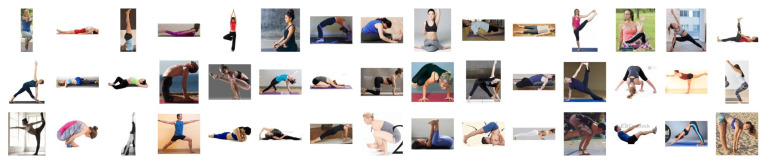
An overview of 45 categories of yoga poses in Dataset A.

**Figure 5:**
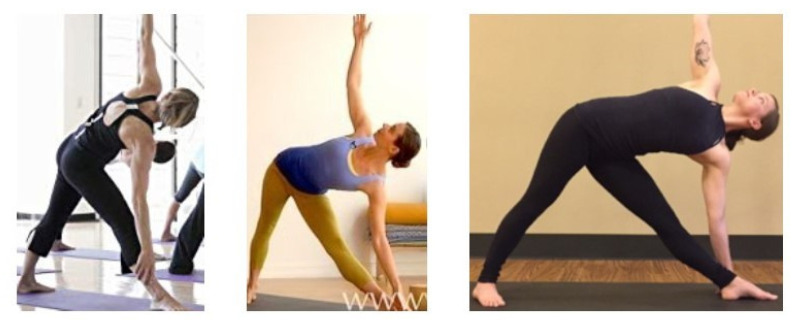
Examples of our yoga pose grading image in Dataset B. Three images are selected from the category *Utthita Trikonasana*. These images have *low*, *medium*, and *high* grades, respectively (from the left to the right).

- *Dataset A*: This is the yoga pose classification image dataset adopted from Kaggle [37], where 45 categories and 1931 images are selected. In this dataset, images are captured with various resolutions and diverse backgrounds. An overview of these categories is illustrated in Figure 4.
- *Dataset B*: This is the yoga pose grading image dataset that we constructed. In this dataset, 3000 triplet examples are collected, where each triplet example consists of three pose images that belong to the same yoga pose category. These images have various resolutions and diverse backgrounds. Then, professional yoga teachers [38] are engaged to grade these three images with respect to the standard pose image in order to obtain three grades: *high*-quality, *medium*-quality, and *low*-quality. An example of this dataset is illustrated in Figure 5.

These two serve as the benchmark datasets for evaluating and justifying the proposed approach in experiments.

### 4.2. Performance Metrics

The performance of the proposed approach is evaluated using the two types of performance metrics below.

The first method is the pose recognition performance evaluation using Dataset A. Two images (simulating one image from the student and the other image from the teacher) are randomly selected from this dataset. Then, the proposed approach is used to evaluate whether their feature similarity is smaller than a user-defined threshold (it is set to 0.75 in our experiments) in order to make a binary decision of whether they belong to the same category. Subsequently, the following four criteria are defined:

- *True positives* (TP): The two input images are from the same category (accurate pose), and the proposed approach correctly classifies them into the same category.
- *False positives* (FP): The two input images are not from the same category (inaccurate pose); however, the proposed approach wrongly classifies them into the same category.
- *True negatives* (TN): The two images are not from the same category (inaccurate pose), and the proposed approach correctly classifies them as different poses.
- *False negatives* (FN): The two images are from the same category (accurate pose); however, the proposed approach wrongly classifies them as different poses.

Based on the four aforementioned criteria, we further define the following performance metrics: *Accuracy*, *Precision*, *Recall*, and *F*1.

(5) $$\begin{array}{ccc} Accuracy & = & \frac{TP+TN}{TP+FP+FN+TN}, \end{array}$$

(6) $$\begin{array}{ccc} Precision & = & \frac{TP}{TP+FP}, \end{array}$$

(7) $$\begin{array}{ccc} Recall & = & \frac{TP}{TP+FN}, \end{array}$$

(8) $$\begin{array}{ccc} F1 & = & 2\times \frac{Precision\times Recall}{Precision+Recall}. \end{array}$$

In this experiment, 1656 pairs of photos are randomly selected from Dataset A, including 828 positive pairs and 828 negative pairs.

The second method is the pose feature similarity performance evaluation using Dataset B. The criterion is: The distance between *high*-quality and *low*-quality pairs should be larger than that between *high*-quality and *medium*-quality pairs, and between *low*-quality and *medium*-quality pairs. The proposed approach is evaluated and its performance *Accuracy* is defined as the ratio between the number of tests where the proposed approach makes the correct decision and the number of total tests. In this experiment, 254 examples from Dataset B are used.

### 4.3. Baseline Approaches

The relevant yoga pose grading works [29,30,31,32] were reviewed in Section 2.2. These approaches are not suitable in our experiments to be able to provide a fair comparison. First, the method in [29] needs to compare the whole pose image, which is different from the proposed approach that uses only skeleton keypoints. Second, the methods in [30,31,32] require domain knowledge to define the rules for checking the angles of the skeleton keypoints of various yoga poses, which is not available for our pose dataset.

In order to conduct a fair experiment to justify the performance of the proposed approach, we define the following two baseline approaches in the performance comparison.

- *Baseline Approach 1*: This extracts the skeleton keypoints from the input pose image and then builds a virtual skeleton image as follows. The size of the skeleton image is first set to (224,224), then the background color is set to black, each keypoint is then assigned a unique color, and the connections between them are drawn according to the definition of the keypoints. In addition, the image augmentation method is used in the model training, including a random rotation of up to 30 degrees, random scaling, and cropping with a factor in the interval between 0.8 and 1.0. The MobileNetV3 network [39] is used as the backbone, the cross-entropy loss is used, and the output feature vector length is 128. In the model training, 1931 images from 45 categories are used. Finally, the encoded features are used to compare feature similarity in the inference process.
- *Baseline Approach 2*: This exploits the same model architecture as the proposed approach. However, the cross-entropy loss is used to build a pose classification model. In the model training, 1931 images from 45 categories are used. After the model is trained using Dataset A, the encoded pose feature is used to compare feature similarity in the inference process.

### 4.4. Implementation Details of the Proposed Approach

The implementation details of the proposed approach are provided as follows. The triplet examples are constructed, as described in Section 3. The Mediapipe [35] is applied on each input yoga pose image to extract its 33 skeleton keypoints. Then, the coordinates of these keypoints from the triplet example are used as the input to the proposed approach. In the model training process, 1931 coarse triplet examples and 591 fine triplet examples are used. The initial learning rate is set to 0.005, with a weight decay of 0.1 to prevent model over-fitting. The coordinates are randomly shifted as augmentation by adding a value randomly drawn from a Gaussian distribution with a zero mean and a 0.02 variance. The stochastic gradient descent optimization algorithm is used with an Adam optimizer [40]. In the proposed triplet loss, the margin $\alpha_{c}$ in (1) is set to 0.1, and both margins $\alpha_{h}$ and $\alpha_{l}$ in (2) are set to 0.2. The model is trained for 300 epochs with a batch size of 256 on the Nvidia Tesla V100 GPU, and with the 1.9.0 version of the PyTorch library.

### 4.5. Experimental Results and Discussions

The first experiment evaluated the performance of the yoga pose grading approach, as shown in Table 2. As seen from this table, the proposed approach is able to achieve the best *Recall* and *F1* performance in Dataset A. In the experiment using Dataset B, the proposed approach is able to achieve the best accuracy performance.

The second experiment is an ablation study to evaluate how the proposed contrastive examples contribute to the final grading performance of the proposed approach. An experiment is conducted to compare the performance of the proposed approach by using the coarse contrastive examples alone and by using both the coarse contrastive examples and the fine contrastive examples, as shown in Table 3. As seen from this table, the proposed approach is able to achieve the best performance using both coarse contrastive examples and fine contrastive examples.

We acknowledge that the proposed approach is not superior to all baseline approaches in terms of the individual performance metric. It is possible to improve the proposed approach in several aspects in future research works. First, more data augmentations can be applied to generate more contrastive pairs, which could further boost the model’s performance in learning the discriminative features of different poses. Second, only the skeleton positions are used in the proposed approach; it would be interesting to incorporate other features, such as the geometrical features (e.g., angular or distance) among skeleton keypoints, into the proposed approach.

In addition, there are several interesting areas that warrant further research to address the limitations of the proposed approach. First, the proposed approach performs automated pose grading for a single image. In practice, yoga learners need to perform a complete cycle to exercise a certain pose. To address this, the proposed approach can be extended to perform yoga pose grading frame by frame. However, it would be interesting to study how such grading could be performed by considering temporal information provided by the learners’ video instead of processing it frame by frame. Second, the proposed approach provides an overall grade for the yoga pose image. It would be interesting to study the quantitative evaluation of the learners’ pose, such as arm angle or distance, so that further interpretable feedback could be provided to improve the motion of the human body in real time.

## 5. Conclusions

A computer vision-based yoga pose grading approach has been proposed in this paper. The proposed approach was able to automatically grade the yoga pose image via the learned contrastive skeleton feature representations. The proposed approach was able to produce more accurate pose grading, as verified in our experimental results with the use of two benchmark datasets.

## Figures and Tables

**Table 1:** An overview of related yoga pose classification and yoga pose grading research works in the literature. “−” means “not applicable”.

Data	Method	Year	Pose Classification	Pose Grading	Number of Pose Categories	Remark
Wearable device-based	[7]	2019	*√*	*√*	18	Neural network and IMU data
	[8]	2021	−	−	−	Pose measurement
	[9]	2014	*√*	−	12	Body contour-based matching
Kinect-based	[10]	2018	*√*	−	6	Adaboost
	[11]	2018	*√*	−	5	Pose-based matching
Computer vision-based	[20]	2019	*√*	−	6	OpenPose + CNN-LSTM for video
	[21]	2019	*√*	−	42	Motion capture image + CNN
	[22]	2019	*√*	−	26	Image-based CNN
	[23]	2020	*√*	−	6	OpenPose + CNN
	[24]	2020	*√*	−	6	Rule-based classification
	[25]	2020	*√*	−	82	Image-based CNN
	[26]	2021	*√*	−	10	Image-based CNN
	[27]	2021	*√*	−	14	Image-based CNN
	[28]	2021	*√*	−	10	3D CNN for video
	[29]	2011	−	*√*	−	Handcrafted SURF feature of the pose image
	[30]	2018	−	*√*	12	Domain knowledge to check skeleton keypoints
	[31]	2021	−	*√*	5	Domain knowledge to check skeleton keypoints
	[32]	2021	−	*√*	21	Domain knowledge to check skeleton keypoints
	Ours	−	−	*√*	45	Contrastive skeleton feature representations

**Table 2:** Yoga pose grading performance comparison. The best performance is indicated by the bold fonts.

Method	Dataset A				Dataset B
	Accuracy	Precision	Recall	F1	Accuracy
Baseline Approach 1	0.7953	**0.9939**	0.5942	0.7438	0.5709
Baseline Approach 2	**0.8327**	0.9911	0.6715	0.8006	0.6004
Proposed Approach	0.8321	0.8819	**0.7669**	**0.8204**	**0.6358**

**Table 3:** The ablation study of how the proposed contrastive examples contribute to the final pose grading performance of the proposed approach. The best performance is indicated by the bold fonts.

Proposed Approach	Dataset A				Dataset B
	Accuracy	Precision	Recall	F1	Accuracy
Coarse contrastive examples only	0.7760	0.6961	**0.9795**	0.8138	0.5827
Both coarse and fine contrastive examples	**0.8321**	**0.8819**	0.7669	**0.8204**	**0.6358**

## Data Availability

Not applicable.
